# Delivering equitable pain care: Lessons from the Scottish Service Model for Chronic Pain

**DOI:** 10.1177/20494637231180485

**Published:** 2023-05-30

**Authors:** Cassandra Macgregor, Christopher Seenan, David N. Blane

**Affiliations:** 1 Department of Physiotherapy and Paramedicine, School of Health and Life Sciences, 150824 Glasgow Caledonian University, Glasgow, UK; 2 NHS Lanarkshire Chronic Pain Service 3077 , Coatbridge, UK; 3 School of Health and Wellbeing, 3526 University of Glasgow, Glasgow, UK

In this editorial, we discuss the challenges of implementing pain care in a way that is relevant and accessible to the person with chronic pain, and also guided by research. We use the Scottish Service Model as an example of strategic delivery of pain care.

Chronic pain is a broad, heterogeneous category of conditions, recognised as either a primary condition, secondary condition associated with another condition such as rheumatoid arthritis or both.^1^ Heterogeneity extends to biological, psychological and social aspects of the condition. Chronic pain is commonly experienced alongside other long-term conditions (multimorbidity).^2^ It is also experienced unequally with higher severity and prevalence in racially minoritised groups, women, and with socioeconomic disadvantage.^3–5^ There is a high societal cost associated with impact on work, benefits claims and healthcare provision;^6,7^ therefore, getting pain care right matters to both the person with pain and to society as a whole. In our current context of rising health and social inequalities, and cost of living crisis, delivering pain care equitably should be a key consideration for strategic implementation.

Research-based guidelines for chronic pain conditions commonly include exercise, cognitive behavioural therapies and medications.^8–10^ In practice, however, pain care often begins with medication prescription, with potential escalation of this leading to concerning levels of opioid and other prescribing, especially prevalent in areas of deprivation within the UK.^11,12^ Experiences of pain care can seem disjointed and invalidating, with over reliance on medical approaches.^13,14^ While sometimes care may be *available*, it is not always *accessible* to the person with pain if they cannot travel to it, take time off work or away from caring responsibilities, perceive it as relevant, or access digital services. So how do we navigate the needed change from the focus on medical interventions, to research-based rehabilitative and multidisciplinary interventions? How do we shift the focus to long-term, quality of life and functional goals, and the way that these can be delivered accessibly?

In practice, exercise, cognitive behavioural therapies and pharmaceutical care are most effectively implemented in combination, over time, and may require input from a range of services and disciplines. Strategic implementation in a coherent way is important, and availability of care is the first step towards access. The Scottish Service Model for Chronic Pain^15^ shows how pain care is stratified at different levels within Scotland (Table 1), governance being different across the four UK nations.^16^

**Table 1.** Scottish Service Model for Chronic Pain.

Population level
The ‘population’ level is also referred to as ‘self-management’^7,15^ which includes services which anyone can use, without referral. This may include leisure, healthy reading books in the library, third sector organisations (e.g. Pain Concern and Pain Association Scotland), websites for pain care and information leaflets in local pharmacies.
Primary care
Most patients receive care for chronic pain in primary care and this can include GPs, physiotherapists, pharmacists, occupational therapists and nurses. Prescribing data from 2002 suggests an estimated 4.6 million GP consultations a year are for pain.^29^
Secondary care
Health boards were supported through a national steering group to employ multidisciplinary specialist chronic pain teams commonly including medical staff, psychology and physiotherapy, supporting the delivery of pain rehabilitation and a pain management programme.^7,15^ Health boards may also offer medical interventions at this level. Occupational therapy, pharmacy and nursing staff may also be employed this level, with scope for development here.
Tertiary care
Tertiary care in Scotland includes the national pain management programme and spinal cord stimulator service.^7^

The strategic, Scottish Service Model has been built upon by the hard work of many colleagues in driving political change.^15^ However, there is still a lot of work to do in delivering equitable pain care and understanding unequal outcomes across Scotland. The recent Pain Management Framework implementation plan^17^ includes improved access to physiotherapy, pharmacy and occupational therapy in primary care. In addition to delivering specialist pain care to patients, another important role of specialist services and national networks is in providing guidance, education and support to these primary care staff groups. The framework plans offer a practical and strategic approach similar to the ideas expressed in the Lancet’s recent ‘rethinking chronic pain’ editorial.^18^ Importantly in Scotland, services are not subject to competitive tendering contracts, as they are in England, with the Scottish Government and health boards retaining responsibility for these services, which we argue benefits the sustainable implementation of strategic care models.

The framework implementation plan aims to assess variation in services and to use examples of best practice to guide developments in Scotland. Equitable access to, and use of, services is an important aspect of this. Given higher severity and prevalence of chronic pain in areas of deprivation, we should expect higher utilisation of services in these areas. However, Moore et al.^19^ reported higher uptake for their group programme from more affluent areas in Glasgow. There is limited understanding of this aspect of service delivery across Scotland; collection of the Scottish Core minimum dataset^20^ combined with analysis of demographics could be useful here.

Another important consideration, which is difficult to capture in a linear care model, is the idea that people living with chronic pain experience a non-linear ‘journey’ (or ecosystem),^21,22^ which includes experiences of care and service utilisation. Healthcare structures and pathways are usually designed by healthy people with their abilities, literacy and capacity in mind.^23^ They don’t always meet the needs of patients, particularly those affected by poverty and trauma, the effects of which can be experienced across generations. This is where trauma-informed care can play a role.^24^ Scotland has made ambitious plans to make public services trauma-informed,^25^ but it is unclear what impact these have had. We advocate approaches to healthcare professional education which emphasise the contextual, and biopsychosocial, nature of pain^26,27^ and the importance of relational continuity.^28^

At a time when the NHS is under significant pressure, we would be ill advised to dismiss the role of ideology and strategic political decisions, or to take the NHS for granted. Political ideology led to the selection of an approach to competitive tendering of services, placing more responsibility on individuals – and providers – with less accountability on higher governance structures and we argue this threatens the sustainability of strategic, and equitable, pain care. In real life, the patient journey is often complex and requires to take place within a supportive, caring and coherent system of care. While not perfect, the Scottish framework with focus on improving all levels of pain care, strategically, is a welcome development, with aims of improving availability of care; an excellent starting point from which to enhance equity.
